# tDCS-EEG for Predicting Outcome in Patients With Unresponsive Wakefulness Syndrome

**DOI:** 10.3389/fnins.2022.771393

**Published:** 2022-06-24

**Authors:** Baohu Liu, Xu Zhang, Yuanyuan Li, Guoping Duan, Jun Hou, Jiayi Zhao, Tongtong Guo, Dongyu Wu

**Affiliations:** ^1^Department of Rehabilitation, Wangjing Hospital of China Academy of Chinese Medical Sciences, Beijing, China; ^2^Graduate School, Tianjin University of Traditional Chinese Medicine, Tianjin, China

**Keywords:** electroencephalogram, transcranial direct current stimulation, prognosis, disorder of consciousness, non-linear dynamics

## Abstract

**Objectives:**

We aimed to assess the role of transcranial direct current stimulation (tDCS) combined with electroencephalogram (EEG) for predicting prognosis in UWS cases.

**Methods:**

This was a historical control study that enrolled 85 patients with UWS. The subjects were assigned to the control (without tDCS) and tDCS groups. Conventional treatments were implemented in both the control and tDCS groups, along with 40 multi-target tDCS sessions only in the tDCS group. Coma Recovery Scale-Revised (CRS-R) was applied at admission. The non-linear EEG index was evaluated after treatment. The modified Glasgow Outcome Scale (mGOS) was applied 12 months after disease onset.

**Results:**

The mGOS improvement rate in the tDCS group (37.1%) was higher than the control value (22.0%). Linear regression analysis revealed that the local and remote cortical networks under unaffected pain stimulation conditions and the remote cortical network under affected pain stimulation conditions were the main relevant factors for mGOS improvement. Furthermore, the difference in prefrontal-parietal cortical network was used to examine the sensitivity of prognostic assessment in UWS patients. The results showed that prognostic sensitivity could be increased from 54.5% (control group) to 84.6% (tDCS group).

**Conclusions:**

This study proposes a tDCS-EEG protocol for predicting the prognosis of UWS. With multi-target tDCS combined with EEG, the sensitivity of prognostic assessment in patients with UWS was improved. The recovery might be related to improved prefrontal-parietal cortical networks of the unaffected hemisphere.

## Introduction

Severe brain injuries often result in prolonged disorders of consciousness (DOC), including vegetative state (VS) or unresponsive wakefulness syndrome (UWS) and minimally conscious state (MCS). The prognostic assessment of DOC patients is not only directly related to medical decision-making by the patient's family and clinicians, but also the basis for further neurological rehabilitation. To date, the clinical evaluation of patients with DOC mainly depends on the doctor's clinical experience and behavior scales. However, behavioral assessments are inevitably subjective and vulnerable to a variety of personal interferences.

Electroencephalogram (EEG) is broadly utilized to predict patient outcome in DOC as a simple, bedside use and low cost tool. Clinically, the degree of brain damage in DOC patients after brain injury is evaluated mainly by EEG assessment. The researches of resting-state EEG revealed that brain networks can predict patient prognosis in DOC (Chennu et al., 2017; Cacciola et al., 2019). Other studies showed that EEG responsiveness can predict the outcome of DOC patients (Li et al., 2015; Schorr et al., 2015, 2016; Johnsen et al., 2017; Stefan et al., 2018). Because the spectrum of EEG malignant categories (suppression, burst-suppression, α and θ coma and generalized periodic complexes combined) is greatly variable (Young, 2000), it is difficult to quantify different EEG features of malignant categories.

Event-related potential (ERP; including N100, MMN, P300 and N400) is currently the most effective method for evaluating prognosis in patients with DOC. Previous findings revealed that ERP has a good predictive value regarding the outcome of patients with MCS (Morlet and Fischer, 2014; Salvo et al., 2015; Henriques et al., 2016; Zhang et al., 2017a).

TMS combined with EEG (TMS-EEG) has the advantage of not relying on the patients to retain a complete sensory pathway, but their ability to understand and execute instructions. Studies have shown TMS-EEG may be effective in evaluating treatment efficacy in patients with MCS (Rosanova et al., 2012; Bai et al., 2016; Ragazzoni et al., 2017).

From the results of the mentioned studies on prognostic judgments of DOC patients, it is clear that relatively satisfactory prognostic outcomes occurred mainly in MCS patients rather than in UWS patients. The reasons might be as follows. (1) It is common for UWS patients to have severe aphasia, cognitive deficits and/or neurobehavioral pathologies such as apathy. The degree of such issues further affects the accuracy of active paradigms; (2) current prognostic judgment is merely based on the instant assessment of DOC at admission, drawing a conclusion about the prognostic results in the future, ignoring prognosis is greatly affected by the treatments, including transcranial direct current stimulation (tDCS).

tDCS represents an important non-invasive neuromodulation technology. Due to minimal side effects, larger stimulation area and simple operation, it has unique advantages in the rehabilitation of brain injuries. Recently, tDCS has been broadly examined for improving the consciousness level in individuals with DOC (Angelakis et al., 2014; Thibaut et al., 2014, 2019a; Cavaliere et al., 2016; Bai et al., 2017b, 2018; Dimitri et al., 2017; Estraneo et al., 2017; Martens et al., 2018; Guo et al., 2019; Zhang Y et al., 2020), especially in some MCS patients. Researchers from different institutions have found that tDCS might improve the prognosis of UWS (Angelakis et al., 2014; Thibaut et al., 2014; Naro et al., 2015; Bai et al., 2017b; Cavinato et al., 2019; Guo et al., 2019; Martens et al., 2019; Wu et al., 2019; Zhang R et al., 2020). The results of Table 1 indicated that the prognosis were improved in a small proportion of UWS patients. It should be pointed out that the positive results were more frequent in patients undergoing electrophysiological assessment rather than behavioral assessment, which was often ignored by researchers (Estraneo et al., 2017; Cavinato et al., 2019).

**Table 1:** Overview of the studies on tDCS for UWS.

**Authors, year**	**Patient**	**Target**	**Sessions**	**Results**
Thibaut et al. (2014)	25 UWS	DLPFC	1	no improvement
Angelakis et al. (2014)	7 UWS	Left S1M1 or DLPFC	5	1 UWS to MCS
Naro et al. (2015)	10 UWS	OFC	1	Unmask such excitability connectivity in some UWS patients.
Bai et al. (2017a)	9 UWS	DLPFC	1	Global cerebral excitability increased
Estraneo et al. (2017)	7 UWS	DLPFC	5	2 EEG changes
Cavinato et al. (2019)	12 UWS	DLPFC	10	showed some local frontal changes in the slow frequencies
Guo et al. (2019)	5 UWS	Precuneus	28	3 showed the CRS-R scores increased
Wu et al. (2019)	8 UWS	DLPFC	10	no improvement
Martens et al. (2019)	4 UWS	Prefrontal	1	no improvement
Zhang et al. (2017b)	5 UWS	DLPFC	10	2 clinically significant improvement
Zhang Y et al. (2020)	15 UWS	Anode centered over the precuneus	28	4 UWS to MCS-

Most UWS patients require long treatment time, which increases the difficulty of determining their prognosis. How to improve the reliability and validity of prognostic judgment and shorten the evaluation period is an important problem that needs to be solved for clinical prognosis judgment in patients with UWS.

Our previous study examining patient prognosis by non-linear dynamics analysis (NDA) of the EEG demonstrated that measuring abnormal interconnections of residual cortical functional islands plays a critical role in predicting prognosis in UWS (Liu et al., 2021). Another study by our group assessing psychomotor inhibition state (PIS) after TBI in cases who recovered from UWS showed that multi-target (prefrontal area, left DLPFC) anodal tDCS could improve PIS (Zhang X et al., 2020). Furthermore, our most recent study confirmed that multi-target tDCS [prefrontal area, left DLPFC and bilateral fronto-temporo-parietal cortices (FTPCs)] could overall improve the prognosis of DOC patients and the prognosis of patients in tDCS group (12/29, UWS to MCS) was significantly better than that in the control group (4/28, UWS to MCS) (Zhang et al., 2021). The above results suggest that multi-target tDCS combined with EEG (tDCS-EEG) is a promising method for predicting the prognosis of UWS patients.

However, there is no currently available protocol for the assessment of UWS by tDCS-EEG. The objectives of this study were: (1) to develop a tDCS-EEG protocol for predicting UWS prognosis; and (2) to investigate the efficiency of tDCS-EEG in predicting the prognosis of UWS. Accordingly, we hypothesized that tDCS could improve the sensitivity of prognostic assessment in UWS patients. To test this hypothesis, multi-target anodal tDCS (prefrontal area, left DLPFC and bilateral FTPCs) was applied. Coma Recovery Scale-Revised (CRS-R) analysis was performed at admission, and the non-linear EEG index was calculated after treatment. The modified Glasgow Outcome Scale (mGOS) was applied 12 months after disease onset (follow-up).

## Materials and Methods

### Patients and Eligibility Criteria

This study was carried out in the Department of Rehabilitation, Wangjing Hospital of China Academy of Chinese Medicine Sciences (CACMS) and Xuanwu Hospital of Capital Medical University. Totally 85 medically stable patients with UWS were enrolled from 2009 to 2019. The subjects consisted of 27 cerebral hemorrhage and 58 brain injury cases, including 61 males and 24 females, aged 17–83 years old. The disease course ranged between 60 and 298 days (median, 121 days). All patients were right-handed, as identified using the Edinburgh Handedness Inventory based on the responses of respective spouses or guardians. Among the 85 patients, 50 recruited from 2009 to 2014 received no tDCS treatment and served as the control group, while the remaining 35 recruited from 2015 to 2019 received multi-target tDCS and served as the experimental group. All patients' spouses or guardians provided signed informed consent. The study design and protocol were approved by the ethics committees of both centers.

Inclusion criteria were: (1) TBI or cerebral hemorrhage; (2) confirmed diagnosis of UWS as defined by the Multi-Society Task Force Report on VS (Ashwal and Cranford, 1995); (3) brain injury course of 2–10 months prior to study participation; (4) no previous history of brain injury.

Exclusion criteria were: (1) unstable condition; (2) epilepsy; (3) locked-in syndrome; (4) primary brain-stem injury; (5) regional skin injury under the tDCS electrode; (6) severe spasticity resulting in obvious electromyography (EMG) artifacts; (7) obvious communicating or obstructive hydrocephalus was present.

### Design and Procedures

**Figure 1:**
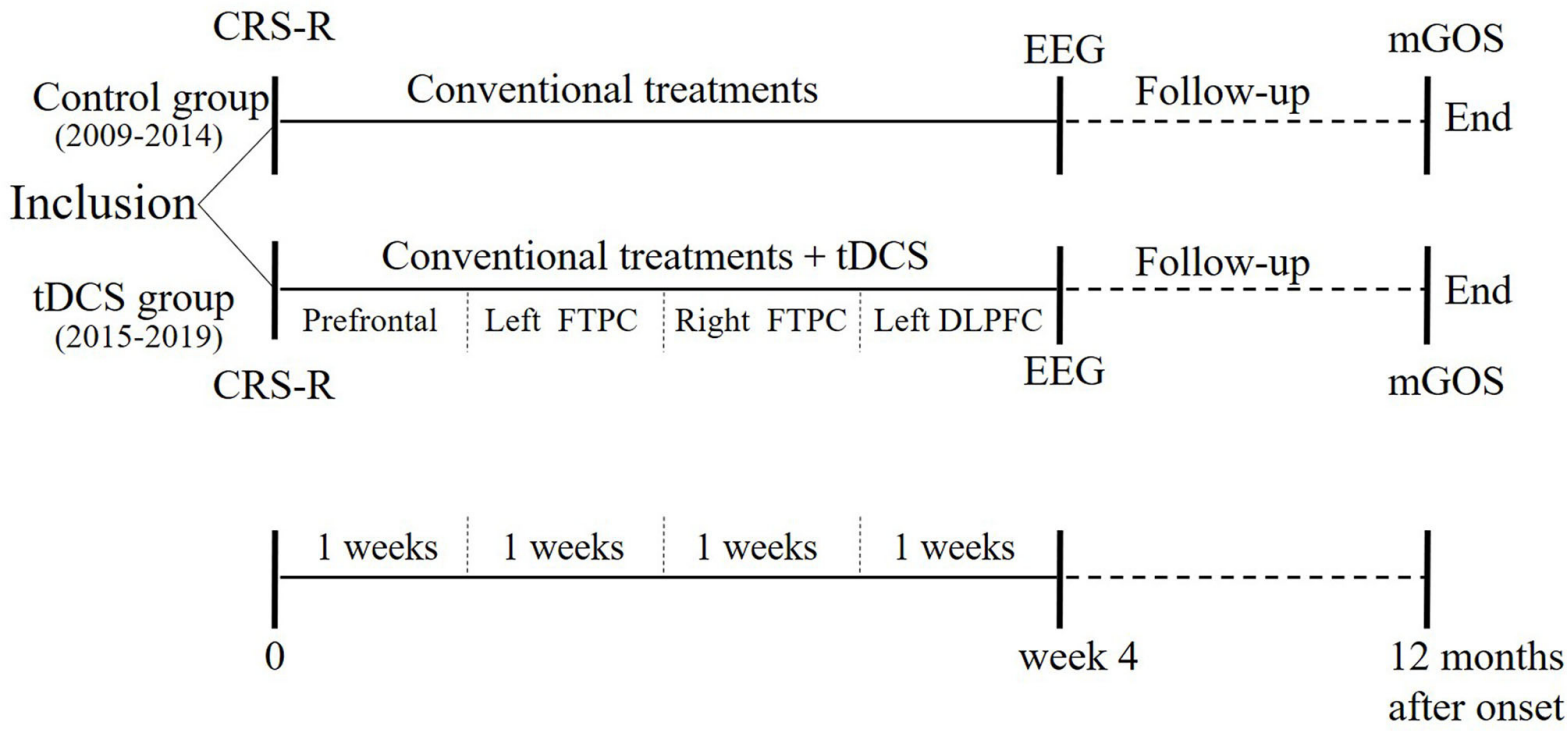
Study protocol.

This was a historical control study. The cases enrolled from 2009 to 2014 were not treated by tDCS, and constituted the control group (historical controls); those recruited from 2015 to 2019 were treated by tDCS as the tDCS group. Conventional treatments were identical in both groups. In the tDCS group, the order of tDCS targets was: prefrontal area, left FTPC, right FTPC and left DLPFC. Each target was treated 2 times/day for 5 days per week. Thus, it took 4 weeks to complete all 4 targets (40 sessions). CRS-R was also applied at admission, and the non-linear EEG index was evaluated after treatment. In addition, the mGOS was applied 12 months after onset. Figure 1 was the protocol of this study.

### Treatments

Direct current delivery utilized an IS200 portable battery-driven device manufactured by Chengdu (China). In this study, 2.0 mA (0.056 mA/cm^2^) for 20 min was applied to prefrontal area and bilateral FTPC utilizing saline-soaked surface sponge electrodes (5 cm × 7 cm); meanwhile, 1.2 mA (0.056 mA/cm^2^) was applied with 4.5 cm × 5 cm electrodes for left DLPFC.

**Figure 2:**
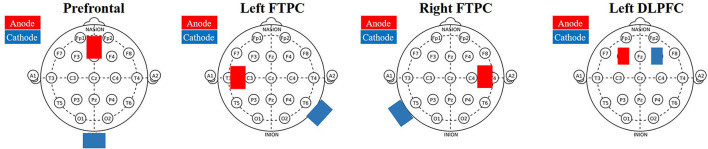
Transcranial direct current stimulation electrodes positioning.

According to our preliminary research and the international 10–20 system, the prefrontal area was identified at 3.5 cm above the FPz; the left DLPFC was located as described previously (O'Neil-Pirozzi et al., 2017). Cathodes for the prefrontal area, left DLPFC and bilateral FTPCs were, respectively, placed over the neck, at F4 and the back of the opposite shoulder (Figure 2).

Routine treatments were administered for 50 min twice a day, including (1) multimodal sensory and auditory stimulation; (2) bedside routine physical therapy including keeping good limb position, chronic stretching and physical approaches and methods; (3) environmental enrichment therapies, e.g., auditory, sensory and visual enrichment therapies.

### Clinical Evaluation

Brain magnetic resonance imaging (MRI) or computed tomography (CT) was carried out in every participant. CRS-R scores were obtained by two examiners with great experience in an independent manner at admission. CRS-R was used on the basis of the highest score recorded at least five successive times by CRS-R in 1 week. mGOS scores were obtained for assessing functional prognosis a year after brain injury. The mGOS had 6 designations, including death, VS, MCS, severe disability, moderate disability and good recovery (Luauté et al., 2010; Li et al., 2015). Improvement (recovery) was defined as mGOS ≥ 3 while no improvement (non-recovery) mGOS ≤ 2.

### EEG Recording and Non-linear Dynamics Analysis

EEG signal recording utilized a wireless 16-channel digital EEG system (ZN16E, Chengdu, China) under closed-eyes and pain stimulation conditions according to the international 10–20 system. For maximizing cortical activation, the acupoints LI4, ST36, LI11, SP6, SJ5, KI1, LR3 and PC6 were electrically stimulated as pain stimuli (starting with the affected side before the unaffected one) with a Han's acupoint nerve stimulator while EEG was being performed. The process of EEG recording and analysis method were as described in previous studies (Wu et al., 2011a; Liu et al., 2021).

Cross-approximate entropy (C-ApEn) was assessed for determining cortical response in DOC patients. The detailed algorithm was described previously (Wu et al., 2011a). C-ApEn was used to analyse two related time series and measure their degree of asynchrony by comparing sequences from one series to those of the second series to reflect the spatial decorrelation of cortical potentials from two remote sites. Higher values of C-ApEn indicated higher degrees of inter-cortical communication or information flow (Wu et al., 2011b). Because the affected sides in patients with brain damage were variable, the affected (A) and unaffected (U) sides were utilized to replace the traditional left and right sides, respectively. Thus, the subscripts of the 16 electrodes were changed to FP_U_, FP_A_, F_U_, F_A_, AT_U_ (anterior temporal), AT_A_, C_U_, C_A_, MT_U_ (middle temporal), MT_A_, P_U_, P_A_, PT_U_ (posterior temporal), PT_A_, O_U_, and O_A_. Local C-ApEn (i.e., C_U_-F_U_, C_U_-MT_U_, C_U_-P_U_, C_A_-F_A_, C_A_-MT_A_ and C_A_-P_A_) and remote C-ApEn (i.e., C_U_-FP_U_, C_U_-O_U_, C_A_-FP_A_ and C_A_-O_A_) were calculated to determine the correlation between C-ApEn changes and defective information transmission.

### Statistical Analysis

SPSS 22.0 (SPSS, USA) was utilized for data analysis. Baseline characteristics between the control and tDCS groups were compared by independent samples *t*-test and the Chi-squared test for continuous and dichotomous variables, respectively. Independent samples *t*-test was performed for quantitative data with normal distribution and homogeneity of variance; otherwise, the Wilcoxon signed-rank test was carried out. Qualitative data were expressed in percentage (%) for mGOS improvement, and assessed by the Chi-squared test. Pearson's correlation analysis was carried out for determining correlations between quantitative indicators; indicators with statistically significant correlation and those professionally considered to have an impact on the mGOS score were included in the multiple linear regression model, to determine the factors influencing the mGOS score. Linear regression analysis was also performed to assess the predictive value of EEG on recovery of consciousness. In regression analysis, C-ApEn and clinical (age, sex, lesion and disease course) parameters were independent variables, with mGOS improvement as the dependent variable. *P* < 0.05 indicated statistical significance. Receiver operating characteristic (ROC) curve analysis was carried out for determining prognostic sensitivity and specificity in patients with UWS.

## Results

Table 2 summarizes the patients' baseline features. There were no significant differences in age, sex, lesion, disease course and CRS-R scores between the control and tDCS groups. No subject withdrew from the study. There were no significant adverse events.

**Table 2:** Demographic characteristics of the patients.

**Variables**	**Control group(n = 50)**	**tDCS group** **(n = 35)**	**t**	***p***
Age (year)^a^	44.88 ± 13.68	50.69 ± 13.05	−1.963	0.053
Sex^b^				
Male	37(74.0%)	24(68.6%)	0.299	0.584
Female	13(26.0%)	11(31.4%)		
Lesion^b^				
Traumatic brain injury	34(68.0%)	24(68.6%)	0.003	0.956
Cerebral hemorrhage	16(32.0%)	11(31.4%)		
Duration (days)^a^	134.28 ± 62.45	111.91 ± 50.72	1.752	0.084
CRS-R ^a^	7.72 ± 1.18	7.57 ± 1.38	0.533	0.595

*CRS-R, Coma Recovery Scale-Revised*.

a *Values in cells are mean ± standard deviation*.

b *Values in cells are frequency (percentage)*.

### Clinical Assessment

The results of mGOS improvement in both control and tDCS groups after 1 year of injury are presented in Table 3. The mGOS improvement rates of the control and tDCS groups were 22.0 and 37.1%, respectively. These results showed that the mGOS improvement rate of the tDCS group was higher than that of the control group after 1 year of injury.

**Table 3:** mGOS improvement.

		**Control group**	**tDCS group**
		**(n = 50)**	**(n = 35)**
mGOS improvement^a^	Yes	11(22.0%)	13(37.1%)
	No	39(78.0%)	22(62.9%)

*mGOS, the modified Glasgow Outcome Scale*.

a *Values in cells are frequency (percentage)*.

### Regression Analysis of C-ApEn

Table 4 summarizes the linear regression analysis of mGOS improvement (≥3). Age, sex, lesion and disease course had no significant correlations with mGOS improvement.

**Table 4:** Regression analysis of C-ApEn in UWS.

**Model**	**Unstandardized coefficient**	**Standardized coefficient**	**t**	***P***	**95% CI**	**Collinearity**			
	**B**	**Std. Error**	**β**			**Lower**	**Upper**	**Tolerance**	**VIF**
(Constant)	2.302	0.488		4.715	0.000	1.289	3.314		
AS Ca-Fa	3.551	4.894	0.141	0.726	0.476	−6.598	13.700	0.202	4.948
**AS Ca-FPa**	8.332	3.407	0.418	2.446	0.023^*^	1.267	15.397	0.263	3.804
AS Ca-Oa	−4.366	2.636	−0.212	−1.656	0.112	−9.833	1.101	0.466	2.145
US Cu-Fu	−4.872	4.154	−0.284	−1.173	0.253	−13.486	3.742	0.131	7.631
US Cu-Pu	−5.318	2.876	−0.331	−1.849	0.078	−11.282	0.646	0.240	4.174
**US Cu-MTu**	3.735	1.513	0.394	2.468	0.022^*^	0.596	6.874	0.302	3.314
**US Cu-FPu**	11.492	3.668	0.760	3.133	0.005^**^	3.884	19.100	0.130	7.666
US Cu-Ou	3.925	2.099	0.264	1.870	0.075	−0.427	8.277	0.384	2.603
Sex	0.205	0.268	0.106	0.763	0.453	−0.352	0.761	0.401	2.491
Age	−0.001	0.007	−0.017	−0.162	0.873	−0.016	0.014	0.695	1.439
Duration	−0.003	0.002	−0.177	−1.468	0.156	−0.007	0.001	0.526	1.901
Lesion	−0.084	0.107	−0.087	−0.789	0.439	−0.307	0.138	0.630	1.587

*UWS, unresponsive wakefulness syndrome; C-ApEn, cross-approximate entropy; AS, affected side; US, unaffected side*.

*Bold text indicated C-ApEn had a significant association with mGOS improvement*.

*Significant codes*.

* *P < 0.05*.

** *P < 0.01*.

Concerning C-ApEn in UWS, C_A_-FP_A_ had a significant association with mGOS improvement under affected pain stimulation conditions; meanwhile, C_U_-MT_U_ and C_U_-FP_U_ showed significant associations under unaffected pain stimulation conditions (Table 4). Based on the above regression analysis combined with our previous series of studies, Cu-FPu plays the most critical role in mGOS. Therefore, we selected the difference in the C-ApEn of Cu-FPu between the pain stimulation state and closed-eyes conditions (abbreviated as the difference in C-ApEn) under unaffected pain stimulation conditions as a standard for further prognostic evaluation.

### Evaluation of the Prognosis of UWS Patients (Sensitivity and Specificity)

**Figure 3:**
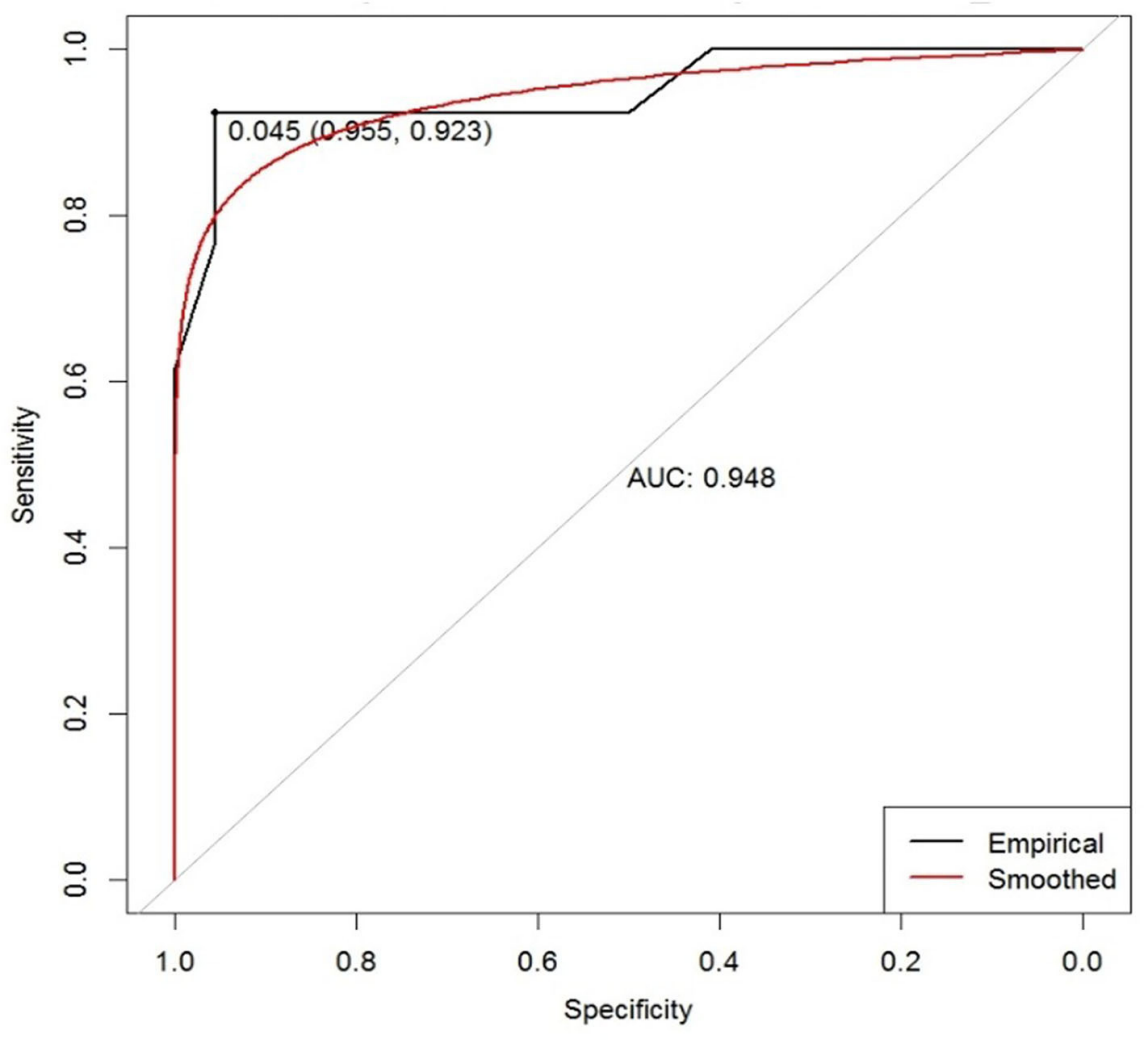
ROC curve based on the difference in the C-ApEn of Cu-FPu under unaffected pain stimulation conditions for predicting patient outcome in UWS. AUC, area under the curve. The empirical line is mapped to the graph according to the actual data, and the smoothed line is the fitted curve. The marked points are the optimal solution given by the system.

ROC curve analysis of the difference in C-ApEn showed an AUC of 0.948 (cut-off = 0.045) (Figure 3). According to the ROC curve analysis combined with our previous studies, we found that when the difference in C-ApEn was 0.07 as the standard, sensitivity and specificity in predicting the outcome of UWS patients were best. Therefore, we set the cut off value to 0.07 instead of 0.045 as the standard for prediction.

Table 5 lists sensitivities and specificities for the prognosis of UWS patients. The results showed that sensitivity in the tDCS group (84.6%) was higher than that of the control group (54.5%), and specificity in both groups remained high (95.5 vs. 100%).

**Table 5:** Sensitivity and specificity of evaluating the prognostic evaluation in of UWS.

	**Control group**	**tDCS group**
Sensitivity^a^	54.5%	84.6%
Specificity^b^	100.0%	95.5%

*tDCS, transcranial direct current stimulation; UWS, unresponsive wakefulness syndrome*.

a *Values in cells are Sensitivity (percentage)*.

b *Values in cells are Specificity (percentage)*.

## Discussion

To date, recovery of UWS patients is still a worldwide challenge and the above routine treatments are unsatisfactory. Currently, surgical intervention such as spinal cord electrical stimulation or deep brain stimulation might be effective in some UWS patients. However, surgical intervention has not been widely recommended by the guidelines, and consensus has not been reached among experts; at present, non-invasive intervention is still the mainstream of clinical practice. All patients in this study were treated non-invasively.

tDCS in combination with electroencephalography (EEG) could help to understand these mechanisms from a neural point of view, some researchers have used the expression of tDCS-EEG before (Roy et al., 2014; Mancini et al., 2016a,b; Vecchio et al., 2021). Different from the online tDCS-EEG or TMS-EEG used to evaluate the instant effect of tDCS or TMS, tDCS-EEG in this study was mainly used to evaluate the curative effect after 1-session tDCS treatment, although it could also provide online cortical effect of tDCS.

To our knowledge, this study was the first to propose a tDCS-EEG protocol for predicting the prognosis of UWS, not based on an instant effect. Compared with the control group, it was found that the tDCS group has a better prognosis improvement effect after 1 year of injury. Moreover, these results confirmed the hypothesis that tDCS could improve the sensitivity of prognostic assessment in patients with UWS.

### Reasons for Designing a Historical Controlled Trial

Unlike retrospective study, a historical controlled trial compares the outcomes of a group of patients currently treated with an intervention with the outcomes of a group of patients previously treated with the same disease but not treated with that intervention, to evaluate the efficacy of that intervention. In this study, among the 85 patients, 50 recruited from 2009 to 2014 received no tDCS treatment and served as the control group, while the remaining 35 recruited from 2015 to 2019 received multi-target tDCS and served as the experimental group.

In terms of study reliability and validity, the randomized controlled trial (RCT) design is the best choice. However, the present trial was designed as a historical control study rather than a RCT because of the following reasons. Firstly, it is difficult to design an RCT involving multi-target tDCS in patients with UWS. According to our preliminary analysis, it takes 4 weeks (40 sessions) to complete the tDCS treatment cycle for all 4 stimulation targets. Secondly, one of the clinical reactions of anodal tDCS is muscle tone elevation, an obvious change that makes it difficult to blind the evaluator. Thirdly, patients with UWS require maximal curative effects in the context of this enormous disability. It is hard to conduct RCTs using sham stimulation. This may be due to ethical issues because of the severity of clinical conditions. Obtaining informed consent from the pessimistic individuals' legal guardians is also a challenge. Therefore, an RCT with sham stimulation was not applied in this study. Instead, a historical control study design was used to compare multi-target tDCS and conventional treatment for prognosis in patients with UWS.

### The Reasons for Choosing Multi-Target tDCS in Patients With UWS

Scientists and clinicians have used tDCS to stimulate brain regions to modulate brain function and diseases for many years. More excitingly, tDCS promotes rehabilitation in individuals with DOC, mainly MCS but not UWS (Keeser et al., 2011; Angelakis et al., 2014; Thibaut et al., 2014, 2017, 2019b; Zhang et al., 2017b; Zhang Y et al., 2020). Although these studies still used single targets to observe the efficacy of tDCS in DOC patients, they provide a reference for the treatment of different target areas and the corresponding research basis.

Many studies and our previous studies have found that prefrontal-parietal cortical networks plays a critical role in improvement in patients with UWS (Cavinato et al., 2015, 2019; Naro et al., 2016a,b; Bai et al., 2017a,c; Cacciola et al., 2019; Zhang et al., 2021). The application of active tDCS on OFC can improve the level of consciousness by improving the residual connectivity between prefrontal lobe and motor area (Naro et al., 2015). Two networks have been identified as potential mediators of consciousness: the default mode network, functionally related to internal awareness (Vanhaudenhuyse et al., 2010; Snyder and Raichle, 2012) and the frontoparietal executive control network, which processes external stimuli (Golland et al., 2007; Vanhaudenhuyse et al., 2011). The stimulated left DLPFC area has central integration function, receives somatosensory and visual input from the parietal heteromodal association cortices, involves motor, visual, tactile and spatial localization, and projects to subcortical cholinergic and monoaminergic sources (Heekeren et al., 2006). A study showed that tDCS over the left DLPFC can increase the functional connectivity between the “default mode” and the bilateral frontal-parietal associative cortical network (Keeser et al., 2011). These studies have provided us with treatment references regarding different target areas and the corresponding research basis. However, clinical treatment is different from scientific research, and the former is more concerned about how to maximize the curative effect. It is well-known that brain regions operate jointly, with inter-region communications via excitatory and inhibitory interactions, in broadly distributed cerebral networks (Grefkes and Fink, 2014; Siegel et al., 2015). Chen's research also showed that stimulating multiple cortical areas rather than a single area is more meaningful for rehabilitation (Chen et al., 2019). Similarly, our recent study focused on the efficacy of multi-target and multi-session tDCS in patients with DOC (Zhang et al., 2021). Therefore, we optimized the treatment strategy in this study, applying multi-target tDCS to the prefrontal area, bilateral FTPCs and left DLPFC in patients with UWS. In order to avoid adverse reactions (i.e., epilepsy and spasticity) due to long-term stimulation, we formulated the following stimulation procedures: prefrontal area, left FTPC, right FTPC and left DLPFC. It should be pointed out that the current study aimed to predict the outcome of patients with UWS rather than to observe efficacy.

As shown in Table 3, the mGOS improvement rate of the tDCS group was 37.1%, which was higher than that of the control group (22.0%). Moreover, as depicted in Table 5, sensitivity in the tDCS group was 84.6 vs. 54.5% in the control group. These results showed that tDCS could improve the sensitivity of prognosis in patients with UWS. In summary, multi-target tDCS stimulation can improve prognostic sensitivity in patients with UWS.

### Prefrontal-Parietal Cortical Networks (Cu-FPu) Have a Critical Function in mGOS Improvement in Patients With UWS

Linear regression analysis showed the local cortical network (Cu-MTu) and remote cortical network (Cu-FPu) under unaffected pain stimulation conditions constituted the factors with tightest associations with mGOS improvement, as well as the remote cortical network (C_A_-FP_A_) under affected pain stimulation conditions (Table 4). Similarly, our previous study assessing NDA-EEG's prediction of prognosis in patients with DOC suggested that Cu-MTu and Cu-FPu under unaffected pain stimulation conditions are closely related to mGOS improvement (Liu et al., 2021). Another study by our group examining PIS treatment by tDCS suggested that improved PIS is related to Cu-FPu under unaffected pain stimulation conditions (Zhang X et al., 2020). These findings were basically consistent with the present results. According to the above regression analysis data combined with our previous series of studies, prefrontal-parietal cortical networks (Cu-FPu) play a critical role in mGOS improvement in patients with UWS. Therefore, we selected the difference in C-ApEn of Cu-FPu under unaffected pain stimulation conditions as a standard for further prognostic evaluation.

### tDCS-EEG Improves Prognostic Sensitivity in UWS Patients

It is well-known that consciousness is not an all-or-nothing phenomenon, and its clinical assessment relies on the doctor's clinical experience and behavior scales (Laureys et al., 2004). In addition, a small number of patients meet the behavioral standards of UWS, but still maintain a certain degree of covert awareness. This can only be done through EEG or the fMRI technology (Monti et al., 2010; Cruse et al., 2011). Moreover, for some UWS patients in clinical practice, even electrophysiology still cannot accurately predict their outcomes. It is precisely because of the above-mentioned difficulties in the prognosis of UWS patients that the misdiagnosis rate of UWS is often astonishingly high (Childs et al., 1993; Schnakers et al., 2009). Accordingly, a highly sensitive evaluation method is particularly important given that although some patients do not show any signs of purposeful behavioral response, they may still recover consciousness after treatment, especially tDCS and TMS cases. Therefore, we used tDCS-EEG for predicting outcome in patients with UWS.

We selected the difference in C-ApEn of Cu-FPu under unaffected pain stimulation conditions as a standard for prognostic evaluation. According to ROC curve analysis (Figure 3) combined with changes in individual differences, we set the cutoff value to 0.07 instead of 0.045. In clinic, we found that the smaller the C-ApEn index, the more likely the interference with various factors would lead to unstable results. On the contrary, the larger the C-ApEn index, the more stable the results. Of course, too large C-ApEn index would reduce the sensitivity of predicting the prognosis of consciousness recovery in patients with UWS. Therefore, considering the above results, we finally set the cutoff value to 0.07; this standard was used to calculate sensitivities and specificities in the tDCS and control groups. This study suggested that prognostic sensitivity could be increased from 54.5% (control group) to 84.6% (tDCS group), with specificity in both groups remaining high (95.5 vs. 100%) (Table 5). The results also indicated that tDCS treatment could dig out more patients with potential recovery. By observing the sensitivity of prognosis in patients with UWS, we can not only judge prognosis more accurately but also avoid missing cases who might recover but are abandoned due to misdiagnosis.

### Limitations

However, there were some limitations in this research. First, although this study had a control group, it was not a standard and rigorous RCT; in future studies, the research protocol and stimulation parameters for tDCS should be optimized. Secondly, from the perspective of diagnostic tests, the sample size was small, and future clinical trials should be carried out based on larger samples. Besides, follow-up was also relatively simple; objective evaluation parameters (e.g., EEG and ERP) should be designed during follow-up in future research. Moreover, future research should pay close attention to multi-session tDCS treatment, because post-traumatic neural plasticity and functional reorganization constitute time-consuming processes.

## Conclusions

We developed a tDCS-EEG protocol predicting UWS prognosis. With multi-target tDCS combined with EEG, the sensitivity of prognostic assessment in patients with UWS was improved. Recovery might be related to improved prefrontal-parietal cortical networks of the unaffected hemisphere.

## Data Availability Statement

The raw data supporting the conclusions of this article will be made available by the authors, without undue reservation.

## Ethics Statement

The studies involving human participants were reviewed and approved by the Ethics Committees of Wangjing Hospital of China Academy of Chinese Medicine Sciences and the Ethics Committees of Xuanwu Hospital of Capital Medical University. Written informed consent to participate in this study was provided by the participants' legal guardian/next of kin.

## Author Contributions

BL and XZ made substantial contributions to data analysis and drafted the manuscript. YL, GD, JH, JZ, and TG treated the patients and acquired the data. DW designed the study, supervised the initial manuscript drafting, and critically revised the manuscript. All authors read and approved the final manuscript.

## Funding

This work was supported by the National Natural Science Foundation of China (Grant Numbers 81171011 and 81572220), the Key Field Project of the 13th Five-Year Plan of the China Academy of Chinese Medical Science (Grant Number ZZ10-015), and the Science and Technology Projects of Beijing (Grant Numbers Z121107001012144 and Z171100001017111).

## Conflict of Interest

The authors declare that the research was conducted in the absence of any commercial or financial relationships that could be construed as a potential conflict of interest.

## Publisher's Note

All claims expressed in this article are solely those of the authors and do not necessarily represent those of their affiliated organizations, or those of the publisher, the editors and the reviewers. Any product that may be evaluated in this article, or claim that may be made by its manufacturer, is not guaranteed or endorsed by the publisher.
